# Test of Specificity in Signalling between Potato Plants in Response to Infection by *Fusarium Solani* and *Phytophthora Infestans*

**DOI:** 10.1007/s10886-024-01521-x

**Published:** 2024-06-21

**Authors:** Lucía Martín-Cacheda, Gregory Röder, Luis Abdala-Roberts, Xoaquín Moreira

**Affiliations:** 1 Misión Biológica de Galicia (MBG-CSIC), Apartado de correos 28, Pontevedra, Galicia, 36080 Spain; 2 Institute of Biology, University of Neuchâtel, Rue Emile-Argand 11, Neuchâtel, 2000 Switzerland; 3 Departamento de Ecología Tropical, Campus de Ciencias Biológicas y Agropecuarias, Universidad Autónoma de Yucatán, Apartado Postal 4-116,, Yucatán Itzimná, 97000. Mérida México

**Keywords:** Specificity, Plant-plant Signalling, Plant-pathogen Interaction, *Fusarium Solani*, *Phytophthora infestans*, *Solanum tuberosum*, Volatile Organic Compounds

## Abstract

Plant-plant signalling via volatile organic compounds (VOCs) in response to insect herbivory has been widely studied, but its occurrence and specificity in response to pathogen attack has received much less attention. To fill this gap, we carried out a greenhouse experiment using two fungal pathogens (*Fusarium solani* and *Phytophthora infestans*) to test for specificity in VOC induction and signalling between potato plants (*Solanum tuberosum*). We paired potato plants in plastic cages, one acting as VOC emitter and the other as receiver, and subjected emitters to one of the following treatments: no infection (control), infected by *F. solani*, or infected by *P. infestans*. We measured total emission and composition of VOCs released by emitter plants to test for pathogen-specificity in VOC induction, and then conducted a pathogen infection bioassay to assess resistance levels on receiver plants by subjecting half of the receivers of each emitter treatment to *F. solani* infection and the other half to *P. infestans* infection. This allowed us to test for specificity in plant VOC signalling by comparing its effects on conspecific and heterospecific sequential infections. Results showed that infection by neither *F. solani* or *P. infestans* produced quantitative (total emissions) or qualitative (compositional) changes in VOC emissions. Mirroring these patterns, emitter infection treatment (control vs. pathogen infection) did not produce a significant change in pathogen infection levels on receiver plants in any case (i.e., either for conspecific or heterospecific sequential infections), indicating a lack of signalling effects which precluded pathogen-based specificity in signalling. We discuss possible mechanisms for lack of pathogen effects on VOC emissions and call for future work testing for pathogen specificity in plant-plant signalling and its implications for plant-pathogen interactions under ecologically relevant scenarios involving infections by multiple pathogens.

**Supplementary Information:**

The online version contains supplementary material available at 10.1007/s10886-024-01521-x.

## Introduction

Plants respond to complex blends of volatile organic compounds (VOCs) emitted by conspecific and heterospecific neighbouring plants (Karban and Karban 2010; Karban et al. 2014). There is mounting evidence that exposure to VOCs from attacked “emitter” plants alerts nearby “receiver” plants, which in turn prime (i.e., prepare) or induce their defences thereby increasing resistance to future attacks (Heil and Karban 2010; Vlot et al. 2021). This form of plant-plant signalling via VOCs has been documented in over 40 species, including several agricultural crops and tree species used in forestry (Heil and Karban 2010; Karban et al. 2014), and in many cases appears to be specific to herbivore- or plant-related factors (Kessler et al. 2023; Moreira and Abdala-Roberts 2019). Such specificity could have key ecological consequences for plant-induced defences and plant-associated interactions, and identifying its causes or drivers can shed light into evolutionary processes shaping plant VOC emissions and the ecological mechanisms underlying plant-plant signalling.

Pathogens are responsible for many types of plant diseases which detrimentally affect plants in both natural and managed ecosystems, and in the latter case represent a major cause of crop loses worldwide (Biere and Goverse 2016; Naz et al. 2022; Strange and Scott 2005). Compared to insect herbivores, less studies have investigated the effects of pathogens on VOC-mediated plant-plant signalling (reviewed by Hammerbacher et al. 2019). As for insects, there is evidence from several systems that VOCs emitted by pathogen-infected plants modulate defensive responses and resulting resistance in neighbouring plants (Quintana-Rodriguez et al. 2015; Riedlmeier et al. 2017; Shulaev et al. 1997; Yi et al. 2009). For example, VOCs emitted by tobacco plants infected with tobacco mosaic virus (Shulaev et al. 1997) and lima bean plants infected with *Pseudomonas syringae* (Yi et al. 2009) enhance defences of neighbouring conspecific plants. However, in other systems, responses appear to be highly variable and, in some cases, no evidence of signalling effects has been found. For example, a recent study found that infection by pathogen *Sclerotinia sclerotiorum* did not affect the total amount or composition of VOCs produced by emitter potato plants, which in turn precluded signalling effects on defences and resistance of neighbouring plants (Moreira et al. 2021). Interestingly, *S. sclerotiorum* infection caused a downregulation of several genes coding for VOC precursors, suggesting that this pathogen suppressed volatile induction, presumably preventing plant-plant signalling (Moreira et al. 2021). Collectively, the observed variability within and among studies suggests plant- and/or pathogen-specificity, but its underlying causes or mechanisms remain poorly understood (Hammerbacher et al. 2019). Further work in other systems is needed for a stronger basis of comparison and generalization, combined with a more detailed and explicit investigation of pathogen- and plant-related factors to unveil the mechanisms underlying variability in interaction outcomes.

Plant induced responses to pathogen attack, including those involving VOCs, can change when plants are under attack by multiple pathogens (Brouwer et al. 2023; Ponzio et al. 2013), potentially leading to different interaction outcomes and contrasting predictions on the specificity, strength, and even direction of plant-plant signalling effects. For example, it has been shown that sequential attacks lead to plant-mediated indirect interactions where early-arriving attackers positively or negatively affect subsequent attackers (Biru et al. 2022; Moreira et al. 2015; Wang et al. 2014). Interaction outcomes are often contingent on the feeding guild or lifestyle of pathogens under scenarios of sequential infection, whereby early attackers associated with the same plant defensive metabolic pathway have a negative effect on subsequent attacker of the same guild (i.e., plant induced resistance to latter), but a positive effect when the subsequent attacker is of a different guild (i.e., induced susceptibility due to interference or so-called crosstalk between pathways) (Erb 2018; Mithöfer and Boland 2008; Pieterse et al. 2009; Thaler et al. 2012). Although effects of sequential pathogen infections on non-volatile plant defences have been well studied (e.g., Spoel et al. 2007; Vos et al. 2015; Quiroga et al. 2023), the consequences of these multiple infections on plant-plant signalling are virtually ignored (a gap that also applies to herbivory research; for case of induction of plant VOC by an early herbivore affecting a subsequent herbivore see: Davidson-Lowe and Ali 2021). That said, studies have reported specificity in pathogen effects on VOC induction and in the effects of these pathogen-induced volatiles on plant defences and resistance on the infected plant (reviewed by Sharifi et al. 2018), possibly setting the stage for specificity in plant-plant VOC signalling. However, to our knowledge there have been no studies testing for VOC-mediated plant-plant signalling in response to attack by multiple pathogens, particularly under scenarios of sequential infections which are a common in natural and managed systems.

Potato (*Solanum tuberosum* L., Solanaceae) is one of the most important crops worldwide, with an annual production of more than 359 million tonnes (FAOSTAT 2020). Over the last decades, threat from pests and diseases on this crop have dramatically increased as a consequence of agricultural intensification, along with the detrimental effects of climate change (Alyokhin et al. 2013). In particular, potato plants are susceptible to a wide variety of diseases that can critically decrease yield, as well as the quality and storability of tubers (Landschoot et al. 2017; Singh et al. 2015). Amongst these, late blight caused by *Phytophthora infestans* and potato wilt caused by *Fusarium solani* are two economically important potato diseases worldwide (Alor et al. 2019; Mehmood et al. 2023; Olivieri et al. 2009). *Phytophthora infestans* is a generalist hemibiotrophic oomycete that produces brown or black spots on the surface of potato leaves and stems and elicits the salicylic acid pathway (Zuluaga et al. 2016). *Fusarium solani*, on the other hand, is a generalist necrotrophic fungus that acts as an important causal agent of wilt in potato plant foliage in the field and potato tuber dry rot during storage (Azil et al. 2021; Moctezuma-Zárate et al. 2013) and elicits the jasmonic acid pathway (Wen-Zhong et al. 2021).

In this study, we carried out a greenhouse experiment with these two fungal pathogens (*F. solani* and *P. infestans*) to test for specificity in VOC induction and plant-plant signalling effects on potato pathogen resistance involving sequential conspecific or heterospecific (i.e., crossed) infections. To this end, we paired potato plants in plastic cages, one acting as VOC emitter and the other as a receiver, and subjected emitters to one of the following treatments: no infection (control), infection by *F. solani*, or infection by *P. infestans*. We measured total emission and composition of VOCs released by emitters belonging to each group to test for pathogen-specificity in VOC induction, and then conducted a pathogen resistance bioassay on receivers by subjecting half of the receiver plants of each emitter treatment to *F. solani* infection and the other half to *P. infestans* infection. This allowed us to test for pathogen-related specificity in plant VOC-mediated signalling and compare different scenarios of sequential infections, namely conspecific and heterospecific infections for each pathogen. Because the studied pathogens elicit different plant defensive pathways, we expected them to induce different VOC amounts and/or emission profiles, and that this would lead to specificity in signalling effects on plant resistance to subsequent infection. Specifically, we predicted that VOC emissions induced by each pathogen would increase resistance of receiver plants against subsequent infection by the same pathogen and reduce receiver resistance against the other subsequent pathogen (i.e., cross-talk hypothesis expanded to VOC-mediated signalling). Alternatively, based on our previous findings with pathogens infecting potato plants (Moreira et al. 2021), it is possible that the pathogens suppress VOC induction, precluding or dampening VOC-mediated signalling effects on plant resistance against subsequent (both conspecific and heterospecific) pathogens. Overall, this study provides a more nuanced understanding of pathogen specificity in VOC-mediated plant-plant signalling and its ecological consequences under realistic scenarios involving multiple pathogen infections.

## Materials and Methods

### Experimental Design

**Fig. 1:**
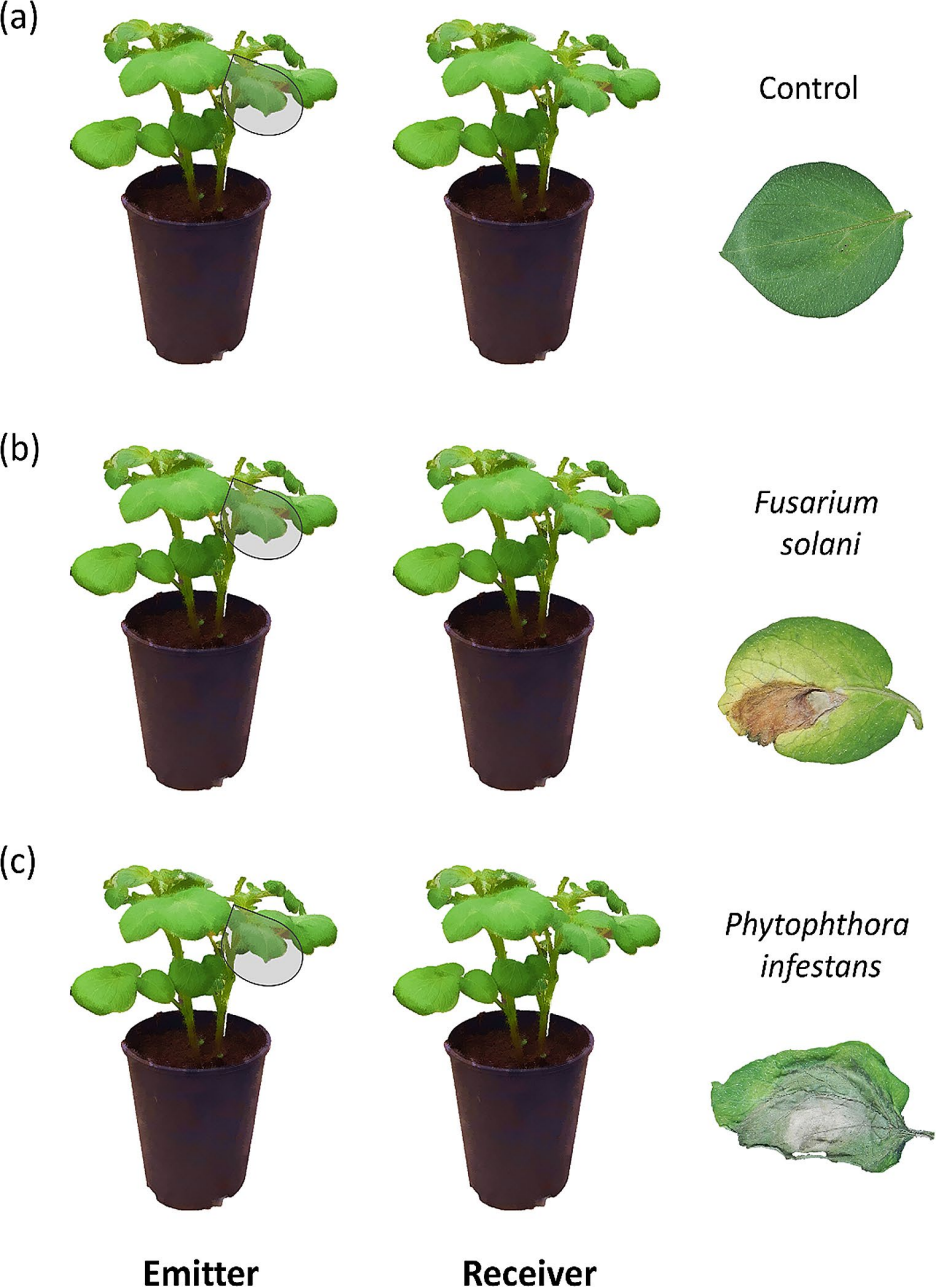
Experimental design used to test for effects of the emitter pathogen infection treatment on plant-plant signalling in potato (*Solanum tuberosum*) plants. We grouped plants in pairs (i.e., one emitter and one receiver), where emitter plants were subjected to one of the following treatments: (**a**) uninfected control, (**b**) infected by *Fusarium solani* and (**c**) infected by *Phytophthora infestans* (*N* = 24)

In May 2023, we individually planted tubers of three potato varieties (*S. tuberosum* L. cultivar Baraka, Desiree, and Monalisa) in 4-L pots containing potting soil with peat (Gramoflor GmbH & Co. KG Produktion, Vechta, Germany) in a glasshouse under controlled light (10 h per day, Photosynthetically Active Radiation = 725 ± 19 µmol m^-2^ s^-1^) and temperature (10 ºC night, 25 ºC day), and were watered twice a week. Before planting, tubers were scrubbed with commercial detergent and running tap-water, and then were surface sterilized with sodium hypochlorite solution (5% available C1^-^) for three minutes and rinsed three times in sterile distilled water to prevent the possible presence of pathogenic microorganisms acquired during storage. Three weeks after planting, we grouped *S. tuberosum* plants of similar size in pairs and placed them in 37.5 × 37.5 × 96.5 cm plastic cages. These cages had two frontal holes covered with a mesh allowing vital airflow. One plant of each pair acted as emitter and the other as receiver (emitter height: 27.3 ± 0.57 cm, receiver height: 22.2 ± 0.58 cm). Within each cage, plants were placed 20 cm apart to avoid physical contact. Adjacent cages were spaced by 2 m to prevent cross-signalling between adjacent replicates (Freundlich et al. 2021). We randomly assigned emitter plants to one of the following: (1) control (intact plants), (2) *F. solani* infection or (3) *P. infestans* infection (Fig. 1). In total, there were 72 cages (24 per treatment) for a total of 72 receiver and 72 emitter plants. For the pathogen infection treatments, we applied three punctures on the upper side of two fully developed leaves using an awl of 1 mm of diameter, and then added agar plugs (0.4 cm in diameter) containing *F. solani* or *P. infestans* mycelia on the punctured site (Moreira et al. 2021). In the case of control plants, we punctured leaves as above and added the agar plugs without pathogen to account for any effects caused by such manipulations. For plants of all experimental groups, we sprayed leaves with sterile distilled water and covered them with transparent plastic bags (10 × 15 cm, polyethylene, K-util) during 48 h to maintain the humidity and promote pathogen infection in treated plants.

### Collection of VOCs Released by Emitter Plants

One week after pathogen inoculation, we removed all emitter plants from cages and collected aboveground VOCs from all the emitters following Rasmann et al. (2011). Briefly, we bagged plants with a 2 L Nalophan bag, and trapped VOCs on a charcoal filter (SKC sorbent tube filled with Anasorb CSC coconut-shell charcoal) for two hours using a Sidekick 224-52MTX pump (0.25 L min^-1^ airflow of technical air N_2_O_2_). We eluted traps with 150 µL dichloromethane (CAS#75-09-2, Merck, Dietikon, Switzerland) to which we had previously added one internal standard (naphthalene CAS#91-20-3, 200 ng in 10 µL dichloromethane). We then injected 1.5 µL of the extract for each sample into an Agilent 7890B gas chromatograph (GC) coupled with a 5977B mass selective detector fitted with a 30 m × 0.25 mm × 0.25 μm film thickness HP-5MS fused silica column (Agilent, Santa Clara, CA, USA). We operated the injection into the GC in pulsed splitless mode (250 ºC, injection pressure 15 psi) with helium as the carrier gas. The GC oven temperature program was: 3.5 min hold at 40 ºC, 5 ºC min^-1^ ramp to 230 ºC, then a 3 min hold at 250 ºC post run (constant helium flow rate 0.9 mL min^-1^). The transfer line was set at 280 ºC. In the MS detector (EI mode), a 33–350 (m/z) mass scan range was used with MS source and quadrupole set at 230 ºC and 150 ºC, respectively. We identified volatile compounds using either commercial pure standards or mass spectrum comparisons with NIST library. In this latter case, Kováts indices calculated relative to the retention times of a series of n-alkanes (C8-C20, Sigma-Aldrich, Merck KGaA, Darmstadt, Germany) analysed under the same chromatographic conditions, were compared with those reported in the literature aiming to improve identification process. Although our Kováts indices matched well with those previously reported, several VOCs should be considered as ‘putative’ until confirmation with standards. We quantified total emission of individual VOCs using normalized peak areas and expressed it as nanograms per hour (ng h^-1^). We obtained the normalized peak area of each individual compound by dividing their integrated peak areas by the integrated peak area of the internal standard (Abdala-Roberts et al. 2022), in order to standardize for variations in the sample volume during the elution process. Reported values for individual VOCs should thus be considered as naphthalene-equivalent nanograms of compound released by each plant per hour. The total emission of VOCs of each sample (i.e., emitter plant) was then obtained by summing the concentrations of individual VOCs. Due to an elution issue, we removed four VOC samples and therefore used 68 samples for statistical analyses (23 control, 23 *F. solani* and 22 *P. infestans* emitter plants).

### Bioassay Testing Induced Resistance in Receiver Plants

The same day after collecting emitter VOCs, we conducted a bioassay on all receiver plants to test whether exposure to VOCs from damaged emitters boosted resistance against pathogen infection and its specificity in relation to the identity of initial vs. subsequent attacker (i.e., conspecific vs. heterospecific indirect pathogen effects via plant VOC signalling). For this, we subjected half of the receiver plants exposed to each group of emitter plants to *F. solani* infection and the other half to *P. infestans* infection. We followed the same procedure as above, using two fully developed leaves. In total, the experiment included 72 plants, corresponding to three initial infection treatments × two receiver infection treatments × 12 replicates per combination. Five days after infection (Rubio-Covarrubias et al. 2005), we sampled infected leaves and photographed them with a Nikon COOLPIX P100 digital camera (10.3 effective megapixels, 26× zoom NIKKOR). Then, we estimated the percentage of damaged area (i.e., with signs of necrosis and chlorosis) due to pathogen infection using ImageJ software (version 1.52a; LOCI, University of Wisconsin, USA).

### Statistical Analyses

*Effects of pathogen infection on emitter VOCs*. We ran general linear mixed models (GLMMs) testing for the effect of emitter pathogen infection treatment (fixed, three levels: control, *F. solani* infection and *P. infestans* infection) on total VOCs released by emitter plants, as well as on each individual compound. We included plant height to account for differences in plant size as a covariate and plant genotype as a random effect, as these sources could potentially affect VOC induced responses. For tests of individual compounds, we performed *P*-value adjustments using the false discovery rate for *P* < 0.05 to avoid inflating Type I error due to multiple testing (Benjamini and Hochberg 1995). In all cases, we used a normal error (identity as link) and log-transformed total VOC emission to achieve normality of residuals.

In addition, we ran a Permutational Multivariate Analyses of Variance (PERMANOVA) with 10,000 permutations to test the emitter treatment effect on VOC composition using individual compound abundances, i.e., qualitative variation in VOC emissions. To visualize these results, we performed a Principal Coordinate Analysis (PCoA) based on Bray-Curtis pairwise dissimilarities and graphed the centroids of each pathogen infection treatment (Moreira et al. 2021). We also identified influential VOCs, i.e., those having the strongest association with the first two ordination axes (R^2^ > 0.60), and displayed these relationships using biplot arrows with the length scaled to R^2^ values.

*Effects of pathogen infection on receiver resistance*. We ran two independent GLMMs testing the effects of emitter infection treatment (fixed, three levels: control, *F. solani* infection and *P. infestans* infection) on the percentage of leaf area damaged by (1) *F. solani* and (2) *P. infestans* on receiver plants. We also included receiver plant height to account for size differences that could affect signalling effects on receiver induced resistance, as well as plant genotype and individual plant as random factors to control for plant genetic variation and the non-independence between leaves sampled for each plant (see sampling methods above), respectively. We log- and square root-transformed percentage of leaf damage caused by *F. solani* and *P. infestans*, respectively, to achieve normality of residuals.

We ran all statistical analyses in R software version 4.3.0 (R Core Team 2020). We implemented general linear mixed models using the *lmer* function from the *lmerTest* package (Kuznetsova et al. 2017). We reported model least-square means and standard errors (back-transformed for log- and square root-transformed data) as descriptive statistics using the *lsmeans* function from the *lsmeans* package (Lenth 2016). Finally, we implemented the PERMANOVA and ordination for VOCs composition using the *adonis* and *capscale* functions respectively, both in the *vegan* package (Oksanen et al. 2016).

## Results

### Pathogen Infection Effects on Emitter Plant VOCs

**Fig. 2:**
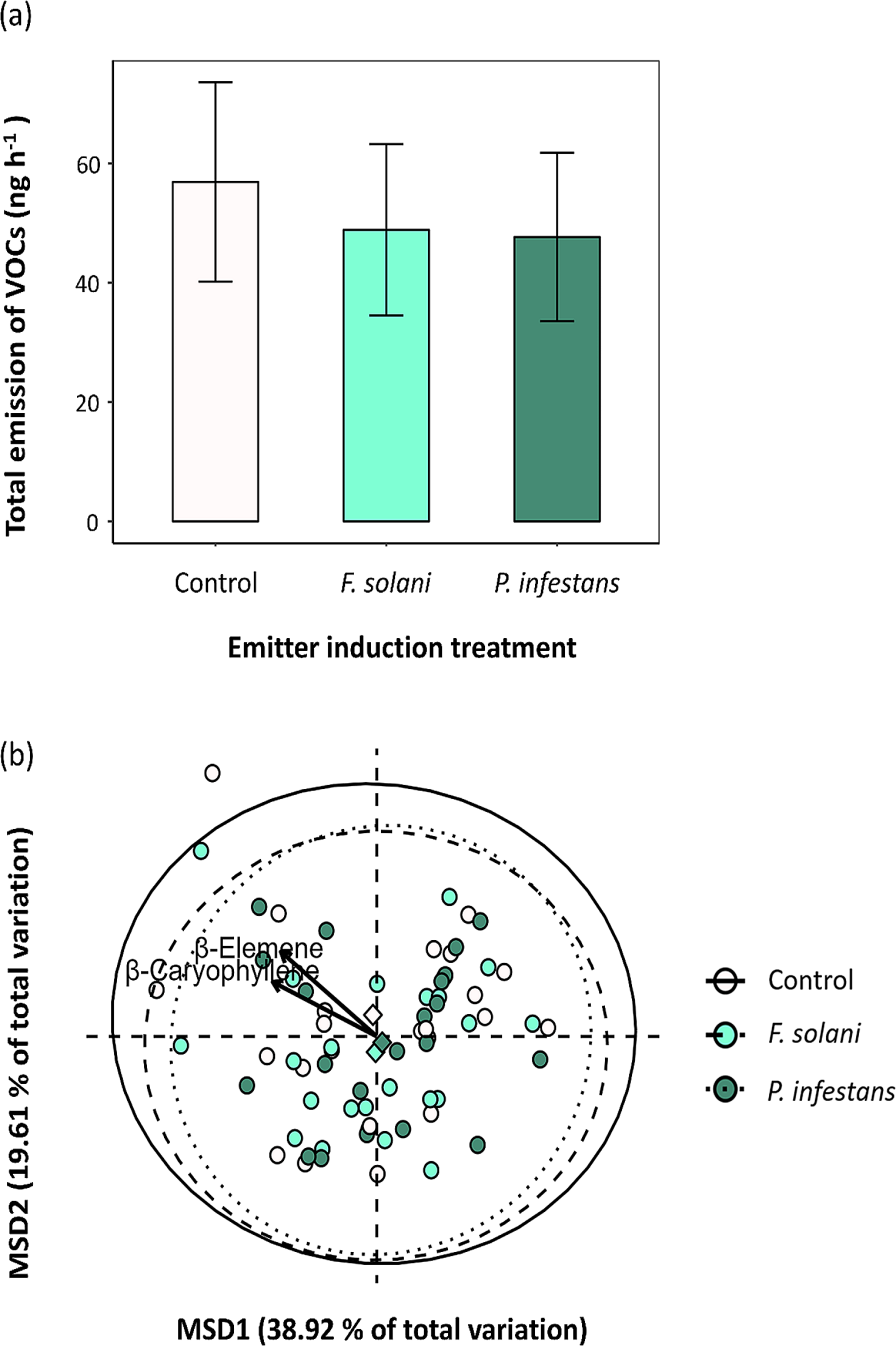
(**a**) Effects of induction treatment (control vs. leaf pathogen infection by *Fusarium solani* and *Phytophthora infestans*) On the total emission of volatile organic compounds (in nanograms per hour, back-transformed from log data) released by emitter potato (*Solanum tuberosum*) plants. Values are back-transformed least-square means ± SE obtained from a general linear mixed model (*N* = 68). (**b**) Unconstrained ordinations showing the effects of emitter induction treatment on log-transformed composition of volatile organic compounds produced by emitter plants. Biplot arrows show associated linear trends with volatiles, scaled to reflect relative magnitude of effects based on R^2^ values (R^2^ > 0.60, *P* < 0.05). The emitter induction treatment ordination displays control and pathogen-induced centroids (represented as diamonds) and 95% ellipses. The first two axes of this ordination accounted for 52.52% of the treatment effect in volatile composition (14.05% and 38.47% respectively). Results are shown in Table 1

We identified a total of 41 VOCs in the headspace of emitter plants (Table S1). The pathogen infection treatment did not significantly affect either total emissions (Table 1; Fig. 2a) or VOC composition (PERMANOVA: Table 1; Fig. 2b). Similarly, analyses of individual compounds showed that there were no differences in emissions between emitter treatments for any of these compounds (Table S1).

**Table 1:** Results from the general linear model testing the effect of induction treatment (three levels: emitters as control, *Fusarium solani* and *Phytophthora infestans* pathogen infection) on the total emission (log-transformed data) and composition of volatile organic compounds (VOCs) released by emitter potato (*Solanum tuberosum*) plants. For VOC composition, we used a permutational multivariate analysis of variance (PERMANOVA) model (using log-transformed data). We included emitters height as a covariate. F values/Pseudo-F, degrees of freedom, and associated significance levels (*P*) are shown

			DF_num, den_	F/Pseudo-F	*P*
Total emission of VOCs			2, 62	0.47	0.63
Composition of VOCs			2, 64	0.49	0.91
Emitter height			1, 63	12.09	< 0.001

### Signalling Effects on Pathogen Resistance in Receiver Plants

**Fig. 3:**
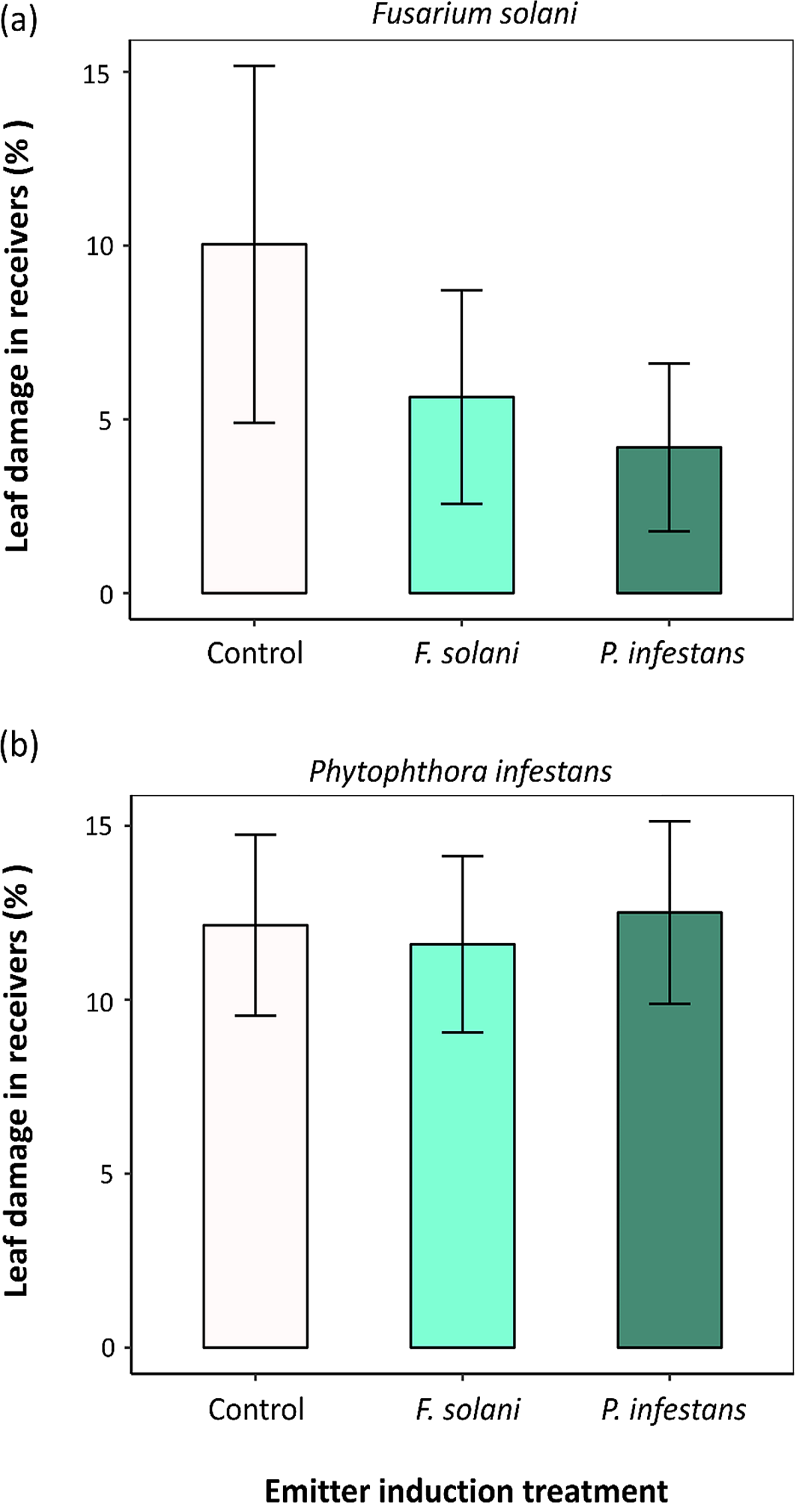
Effect of emitter induction treatment in the emitter *Solanum tuberosum* plants (three levels: emitters as control, *Fusarium solani* and *Phytophthora infestans* pathogen infection) on the percentage of leaf damaged area caused by (**a**) *F. solani* and (**b**) *P. infestans* infection on receiver potato plants. Bars are back-transformed least square means ± SE obtained from the corresponding linear model (*N* = 12). Results are shown in Table 2

Consistent with VOC results, analyses indicated that the emitter pathogen infection treatment did not have a significant effect on the percentage of leaf area damaged by either *F. solani* or *P. infestans* on receiver plants (Table 2; Fig. 3).

**Table 2:** Results from a general linear model testing the effect of induction treatment on emitter potato (*Solanum tuberosum*) plants (three levels: emitters as control, *Fusarium solani,* and *Phytophthora infestans* pathogen infection) on the percentage of leaf damaged area caused by *F. solani* and *P. infestans* infection for receiver potato plants. We included receiver height as a covariate. F values, degrees of freedom, and associated *P*-values obtained from the corresponding models are shown

	Fusarium solani			Phytophthora infestans		
	DF_num, den_	F	*P*	DF_num, den_	F	*P*
Treatment	2, 30	2.05	0.15	2, 32	0.03	0.97
Receiver height	1, 30	1.52	0.23	1, 32	0.13	0.72

## Discussion

Past work has shown that potato VOC induction due to feeding by insect herbivores can be highly specific as a function of enemy identity. For example, damage by the specialist Colorado potato beetle *Leptinotarsa decemlineata* did not induce VOCs and this presumably precluded signalling effects (Abdala-Roberts et al. 2022), whereas herbivory by the generalist beet army worm *Spodoptera exigua* drove significant changes in emissions which resulted in signalling effects (Martín-Cacheda et al. 2023a, b; Vázquez-González et al. 2022, 2023; see also work by Davidson-Lowe and Ali 2021). In the case of pathogens, potato induced defences and resistance in response to infection by multiple pathogens was studied recently by our group (Quiroga et al. 2023; for other plants see: Spoel et al. 2007; Vos et al. 2015), but no previous work has tested for effects on VOC emissions (see Ponzio et al. 2013) and resulting plant-plant signalling. We show that neither *F. solani* or *P. infestans* infection produced quantitative (total emissions) or qualitative (compositional) changes in potato VOC emissions, which precluded signalling effects on plant resistance. These findings are inconsistent with past studies with other plant species reporting effects of pathogen infection on VOC emissions in turn leading to heightened resistance in neighbouring plants (Quintana-Rodriguez et al. 2015; Riedlmeier et al. 2017; Shulaev et al. 1997; Zhang et al. 2019), We next discuss these findings in light of several considerations.

First, methodological aspects related to the timing of VOC collection and the duration of the receiver exposure period must be considered. We collected VOCs seven days after pathogen inoculation, following from previous work reporting that leaf damage caused by *P. infestans* on different potato cultivars (e.g., Malinche, Tollocan, Atlantic) exhibits a sharp increase after the fifth day of inoculation (Rubio-Covarrubias et al. 2005). That said, Laothawornkitkul et al. (2010) instead found that leaf infection by *P. infestans* in potato Agria cultivar significantly increased potato emission of several volatiles (e.g., (E)-2-hexenal, 5-ethyl-2(5 H)-furanone and benzene-ethanol) three to four days after inoculation, and differences were not detected two days after inoculation. Given this variability in VOC induction speed (and/or strength), we cannot reject the possibility that VOC induction took place (and peaked) prior to volatile sampling, and as a result we were not able to detect it. In any case, these results call for further tests involving multiple VOC sampling points combined with molecular or physiological responses (including defensive gene expression levels associated with priming; as in Moreira et al. 2021) to achieve a more robust understanding of pathogens effects (or lack of thereof) on potato VOCs.

Second, previous work has shown that necrotrophic pathogens often produce toxins that interfere with signal transduction pathways or inhibit the synthesis of defence-related compounds such as VOCs (reviewed by Shao et al. 2021), possibly explaning the lack of VOC induction. For instance, the necrotrophic fungus *Botrytis cinerea* was found to alter redox processes that increase plant susceptibility to this pathogen (reviewed by Lyon et al. 2007) or elicit the SA (not JA as most necrotrophs) signalling pathway which antagonized with the JA signalling pathway and allowed the fungus to infect tomato plants (El-Oirdi et al. 2011; Sarmento et al. 2011; for examples with insects such as whitefly and spider mites see: Zhang et al. 2009). Likewise, previous work has shown that *P. infestans* and *F. solani* are able to suppress host defences and promote pathogen susceptibility on several crops (Leesutthiphonchai et al. 2018; Tahmasebi et al. 2023). A recent study on potato by our group supports this: receiver plants exposed to VOCs released by *S. sclerotiorum*-infected (vs. control) emitters did not increase their resistance to this pathogen, presumably due to inhibition of plant defence induction (Moreira et al. 2021). This finding is line with results for conspecific sequential infection by this pathogen. It is important to note that *P. infestans* shifts from a biotrophic to necrotrophic lifestyle approximately one week after infection (Grenville-Briggs and Van West 2005), leading to changes in plant signal-transduction pathways and, as consequence, possibly also volatile induction (Ponzio et al. 2013). Because we measured emitter VOCs and conducted the receiver bioassay one week after inoculating emitters with *P. infestans*, it is possible that the pathogen had already shifted to a necrotrophic lifestyle at this time. This would have led to cross-resistance against *F. solani* assuming a necrotrophic-associated plant response predominated at the time of sampling (rather than induced susceptibility, as originally predicted), but such response was not observed either. Further work is warranted testing for VOCs induction and signalling effects at different time points post-induction by this pathogen.

Third, it is important to point out that while leaf infections have been used to induce plant defences, in the case of *F. solani* (e.g., Villarino et al. 2019; Chen et al. 2021) in which aerial infections to leaves can take place, this pathogen is typically a soil-borne microbe that affects potato tubers (Azil et al. 2021). The methodological approach chosen could therefore limit the generality of our results regarding this interaction and its consequences for potato signalling, calling for future work involving leaf and tuber infections and measuring defensive responses in both organs. Accordingly, a previous synthesis indicates within-plant variability in sequential infection outcomes (Moreira et al. 2018), which supports this recommendation.

As a closing note, it is important to consider that we are not able to determine whether pathogen infection for the resistance bioassay influenced receiver defence induction (including the possibility of defence manipulation by the pathogens). Although the lack of pathogen induction of plant VOCs would preclude any such receiver-associated pathogen effects, we call for further work involving measurements of receiver defence both before and after their infection to tease apart emitter VOC-related from receiver-related pathogen effects shaping signalling outcomes. The measurement of plant defences and associated gene expression levels associated to different plant signalling pathways would be particularly useful under scenarios of multiple pathogen attacks in order to yield mechanistic insight into plant defensive traits primed or induced by VOCs and potential response specificity.

### Outlook

More studies on plant signalling in response to multiple attackers are needed, involving pathogens, herbivores, or both, to test and describe a potentially broader range of interactions outcomes and mechanisms underlying plant responses from defence suppression to synergisms. Doing so will also allow to test whether patterns are consistent or differ from interaction patterns involving non-volatile-mediated plant defences. Importantly, the identification of plant VOC receptors (Loreto and D’Auria 2022; Wang and Erb 2022; Widhalm et al. 2023) will represent a key step forward to understand the biochemical underpinnings of specificity in plant VOC signalling effects in response to attack by single enemies to then build from this to understand more complex outcomes involving multiple attackers.

## Electronic Supplementary Material

Below is the link to the electronic supplementary material.

Supplementary Material 1

## Data Availability

Not applicable.
