# Supplementary material for: Test of Specificity in Signalling between Potato Plants in Response to Infection by Fusarium Solani and Phytophthora Infestans
Source: J Chem Ecol. 2024 Jun 21;50(9-10):562–72. doi: 10.1007/s10886-024-01521-x (PMC11493820; doi:10.1007/s10886-024-01521-x)
Supplement: Supplementary file 1 — Supplementary Material 1 [file 10886_2024_1521_MOESM1_ESM.docx]

**Table S1.** Means (± SE) for emission of individual volatile organic compounds (naphthalene-equivalent ng h^-1^) identified by GC-MS under three emitter infection damage treatments (control vs. infection damage by *Fusarium solani* or *Phytophthora infestans*) in *Solanum tuberosum* (potato) plants^1^. RT = Retention times. KRI = Kovats retention index used for identification of compounds without commercial standards (KRI_c_ for calculated values and KRI_e_ for expected values from the NIST database).

| Compound | RT | KRIe | KRIc | Control | *F. solani* | *P. infestans* | *P*-value |
| --- | --- | --- | --- | --- | --- | --- | --- |
| 2-Hexanol† | 5.901 | 801 | 804 | 2.95 ± 0.176 | 2.96 ± 0.16 | 3.31 ± 0.19 | 0.31 |
| Tricyclene | 9.041 | 925 | 921 | 0.99 ± 0.11 | 1.01 ± 0.1 | 1.05 ± 0.12 | 0.90 |
| α-Pinene† | 9.399 | 937 | 934 | 2.53 ± 0.49 | 2.37 ± 0.5 | 2.27 ± 0.46 | 0.96 |
| Sabinene | 10.568 | 974 | 978 | 0.49 ± 0.28 | 0.21 ± 0.04 | 0.19 ± 0.04 | 0.54 |
| β-Pinene | 10.632 | 979 | 980 | 1.41 ± 0.25 | 1.28 ± 0.25 | 1.26 ± 0.29 | 0.88 |
| β-Myrcene† | 11.032 | 991 | 995 | 1.81 ± 0.29 | 1.75 ± 0.25 | 1.95 ± 0.45 | 0.87 |
| 3-Carene† | 11.635 | 1011 | 1016 | 0.84 ± 0.17 | 0.80 ± 0.16 | 0.78 ± 0.15 | 0.99 |
| Limonene† | 12.114 | 1030 | 1033 | 0.45 ± 0.09 | 0.37 ± 0.05 | 0.35 ± 0.05 | 0.80 |
| Eucalyptol† | 12.204 | 1032 | 1036 | 0.79 ± 0.05 | 0.82 ± 0.04 | 0.82 ± 0.05 | 0.78 |
| Linalool† | 14.051 | 1099 | 1100 | 0.76 ± 0.09 | 0.77 ± 0.07 | 0.71 ± 0.11 | 0.66 |
| Nonanal† | 14.187 | 1104 | 1105 | 2.31 ± 0.74 | 1.31 ± 0.39 | 1.79 ± 0.53 | 0.74 |
| 1,3,7-Nonatriene, 4,8-dimethyl-, | 14.477 | 1116 | 1117 | 5.10 ± 1.58 | 3.88 ± 1.49 | 5.25 ± 2.13 | 0.72 |
| Butanoic acid, 3-hexenyl ester, (Z)- | 16.253 | 1187 | 1188 | 0.10 ± 0.04 | 0.13 ± 0.04 | 0.08 ± 0.03 | 0.62 |
| Methyl salicylate | 16.487 | 1192 | 1196 | 0.11 ± 0.05 | 0.06 ± 0.03 | 0.07 ± 0.03 | 0.64 |
| Dodecane† | 16.567 | 1200 |  | 0.98 ± 0.08 | 0.95 ± 0.08 | 0.96 ± 0.11 | 0.94 |
| Decanal | 16.732 | 1206 | 1207 | 0.48 ± 0.13 | 0.34 ± 0.08 | 0.41 ± 0.12 | 0.90 |
| Tridecane† | 18.928 | 1300 |  | 0.84 ± 0.08 | 0.85 ± 0.08 | 0.79 ± 0.01 | 0.79 |
| δ-Elemene | 19.835 | 1338 | 1340 | 0.14 ± 0.073 | 0.13 ± 0.06 | 0.13 ± 0.07 | 0.98 |
| α-Cubebene | 20.11 | 1351 | 1353 | 0.81 ± 0.09 | 1.19 ± 0.29 | 1.03 ± 0.24 | 0.85 |
| α-Copaene† | 20.689 | 1376 | 1379 | 0.61 ± 0.18 | 0.41 ± 0.10 | 0.58 ± 0.26 | 0.85 |
| β-Elemene | 21.019 | 1391 | 1394 | 1.29 ± 0.49 | 1.06 ± 0.34 | 1.18± 0.44 | 0.97 |
| Tetradecane† | 21.145 | 1400 |  | 0.76 ± 0.05 | 0.78 ± 0.09 | 0.84 ± 0.11 | 0.89 |
| α-Gurjunene | 21.363 | 1409 | 1409 | 1.93 ± 0.18 | 1.91 ± 0.15 | 2.19 ± 0.25 | 0.64 |
| β-Caryophyllene† | 21.678 | 1419 | 1423 | 17.06 ± 4.57 | 15.29 ± 3.83 | 16.75 ± 6.83 | 0.98 |
| β-Copaene† | 21.878 | 1432 | 1432 | 0.14 ± 0.07 | 0.10 ± 0.05 | 0.11 ± 0.04 | 0.96 |
| α-Bergamotene | 21.965 | 1435 | 1436 | 0.73 ± 0.25 | 0.64 ± 0.18 | 0.798 ± 0.21 | 0.88 |
| cis-β-Farnesene | 22.115 | 1444 | 1443 | 2.79 ± 0.91 | 2.08 ± 0.51 | 3.72 ± 1.24 | 0.72 |
| (E)-β-Famesene† | 22.349 | 1457 | 1454 | 2.97 ± 1.18 | 2.11 ± 0.77 | 1.83 ± 0.39 | 0.99 |
| β-Santalene | 22.492 | 1462 | 1459 | 0.32 ± 0.08 | 0.36 ± 0.08 | 0.23 ± 0.06 | 0.50 |
| γ-Gurjenene | 22.843 | 1473 | 1475 | 0.63 ± 0.14 | 0.56 ± 0.13 | 0.67 ± 0.22 | 0.94 |
| Germacrene D | 22.971 | 1481 | 1481 | 4.51 ± 2.02 | 3.79 ± 1.52 | 3.69 ± 1.41 | 0.99 |
| β-Selinene | 23.084 | 1486 | 1486 | 2.05 ± 0.56 | 1.97 ± 0.69 | 3.37 ± 1.46 | 0.85 |
| (Z,E)-α-Farnesene | 23.164 | 1490 | 1490 | 1.34± 0.52 | 1.13 ± 0.37 | 0.99 ± 0.24 | 0.92 |
| Bicyclogermacrene | 23.284 | 1495 | 1495 | 0.97 ± 0.25 | 0.95 ± 0.19 | 1.31 ± 0.42 | 0.99 |
| α-Farnesene† | 23.416 | 1508 | 1506 | 0.18 ± 0.08 | 0.02 ± 0.02 | 0.04 ± 0.02 | 0.07 |
| β-Bisabolene | 23.461 | 1509 | 1508 | 0.60 ± 0.15 | 0.58 ± 0.12 | 0.71 ± 0.24 | 0.99 |
| β-Cadinene | 23.627 | 1518 | 1517 | 0.41 ± 0.19 | 0.24 ± 0.08 | 0.22 ± 0.08 | 0.74 |
| β-Sesquiphellandrene | 23.759 | 1524 | 1524 | 3.19 ± 1.66 | 2.53 ± 0.96 | 2.23 ± 0.58 | 0.96 |
| (-)-Globulol | 24.86 | 1581 | 1581 | 6.09 ± 2.08 | 4.14 ± 1.67 | 6.39 ± 2.96 | 0.89 |
| Viridiflorol | 25.045 | 1591 | 1590 | 0.83 ± 0.21 | 0.74 ± 0.11 | 0.69 ± 0.11 | 0.94 |
| Shyobunol | 27.089 | 1701 | 1701 | 1.01 ± 0.22 | 0.72 ± 0.09 | 0.86 ± 0.09 | 0.34 |
| n-Hexadecanoic acid | 31.584 | 1968 | 1964 | 6.82 ± 4.45 | 2.59 ± 2.24 | 0.27 ± 0.07 | 0.19 |

^1^We performed *P*-value adjustments using the False Discovery Rate for *P* < 0.05 to avoid inflating Type I error due to multiple testing.

^†^Compounds identified with commercial pure standards.
